# Supplementary material for: Optimizing electrode placement and information capacity for local field potentials in cortex
Source: Neuroimage. Author manuscript; Available in PMC 2026 Jul 16. (PMC13373879; doi:10.1016/j.neuroimage.2026.121747)
Supplement: 1 [file NIHMS2189794-supplement-1.docx]

**Supplemental Data**


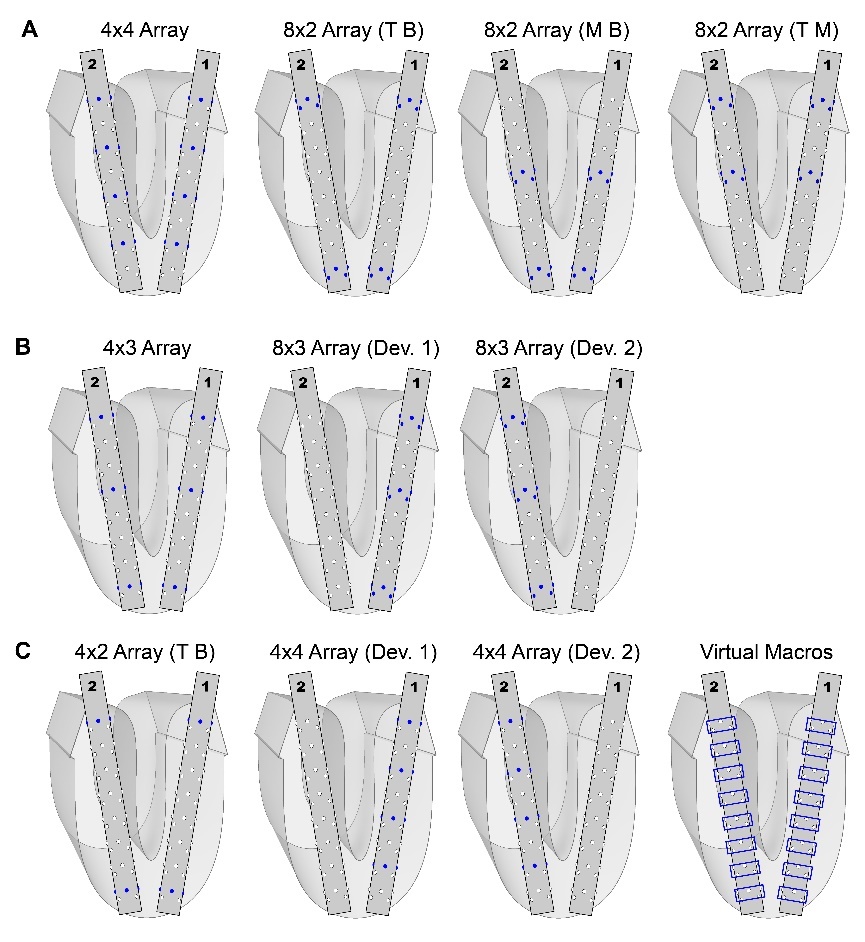


**Supplemental Figure 1.** Demonstration of standard array arrangements using two DiSc devices. By row, these devices contain (A) 32, (B) 24, or (C) 16 active sensors. Classification results from these arrangements are given in Supplemental Table 1.

| 32 Sensors | SEPIO | 4x4 Array | 8x2 Array,  T B | 8x2 Array,  M B | 8x2 Array,  T M |
| --- | --- | --- | --- | --- | --- |
| Simulation Accuracy: | 84% | 83% (-1%) | 71% (-13%) | 70% (-14%) | 80% (-4%) |
| Phantom Accuracy: | 97% | 93% (-4%) | 70% (-27%) | 82% (-15%) | 95% (-2%) |
| **24 Sensors** | SEPIO | 4x3 Array, Both | 8x3 Array,  Dev 1 | 8x3 Array,  Dev 2 | - |
| Simulation Accuracy: | 83% | 76% (-7%) | 45% (-38%) | 44% (-39%) | - |
| Phantom Accuracy: | 95% | 89% (-6%) | 71% (-24%) | 68% (-27%) | - |
| **16 Sensors** | **SEPIO** | **4x2 Array,**  **T B** | 4x4 Array,  Dev 1 | 4x4 Array,  Dev 2 | **Virtual Macros, Both** |
| Simulation Accuracy: | **75%** | **59% (-16%)** | 62% (-13%) | 58% (-27%) | **14% (-61%)** |
| Phantom Accuracy: | **83%** | **55% (-28%)** | 71% (-12%) | 73% (-10%) | **11% (-72%)** |

**Supplemental Table 1.** SEPIO accuracy results for several default array designs visualized in supplemental figure 1. In these comparisons, 32, 24, or 16 sensors are allowed for either standard arrays or as a limit for SEPIO to choose from the total 128 sensors across two DiSc. Percentile accuracy is given with parentheticals containing the change in percentile accuracy relative to the accuracy of SEPIO. Simulation and phantom are kept as separate datasets. All array accuracies are done in Monte-Carlo fashion. Bold and underlined values are displayed in Figure 14.


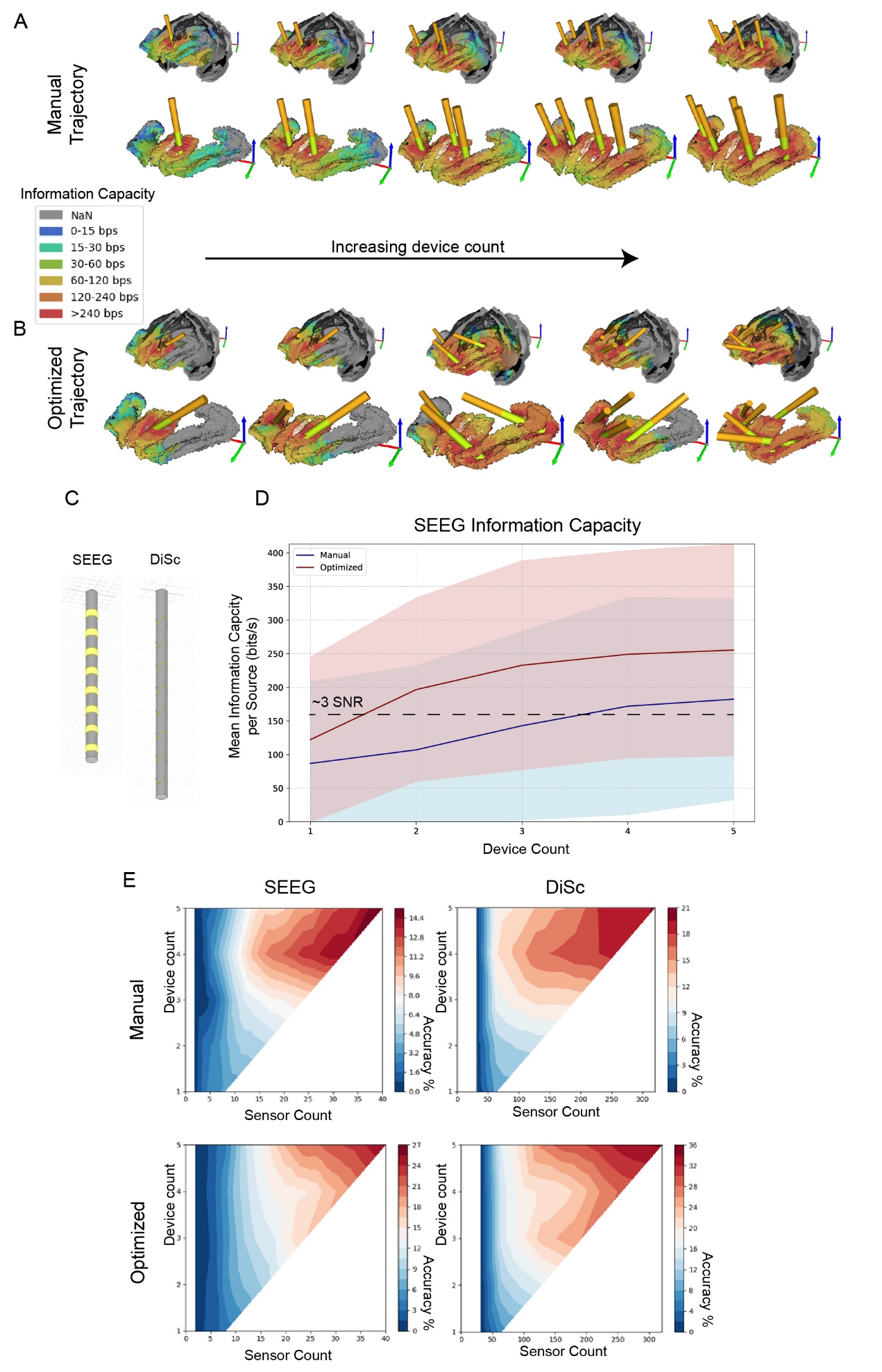


**Supplemental Figure 2.** Case study in macaque anterior cingulate cortex (ACC) and dorsolateral prefrontal cortex (dlPFC) using SEEG and DiSc. (A&B) Bilateral ACC+dlPFC trajectories are visualized with an information capacity heatmap. One to five 8-channel SEEG are shown with the recording probe (yellow) and rigid backend (orange) visualized relative to the ROI and whole cortex. (A) Manual trajectories were placed similar to published literature while (B) optimized trajectories were derived from our software. A heatmap of information capacity depicts the maximum potential recording value for each point on the cortex. Training was performed with the ACC weighed twice (per source) that of the dlPFC to encourage greater central coverage. (C) The two device models have a comparable profile and total span, viewed in ANSYS. (D) Mean information capacity across the ACC+dlPFC ROI for each device count in mean and optimized trajectories. Values are calculated on the optimization test dataset. Mean lines are shown with shaded regions depicting one standard deviation. A dashed line is added at a level depicting roughly 3 SNR signal acquisition. (E) SEPIO heatmaps comparing trajectories and devices. Heat map range and sensor count varies between SEEG and DiSc. Percent accuracy is determined from SEPIO classification accuracy on test data withheld during model training. 13,658 unique sources and classes yield chance accuracy below 0.01%.

**Notes on Supp. Fig. 2:** This ACC-dlPFC ROI provides a much larger, diverse, and difficult testing environment for trajectory optimization. Device orientation diversity improves the potential signal measurement and diversity when source orientations vary. This is observed in all optimized trajectories with two or more devices. Often, these inter-device angles vary by $20^{\circ}$ or more, occasionally becoming nearly orthogonal. Since this algorithm considers device spacing and potential collision of the device backend, the distribution tends to follow directional trends with clusters of devices pointing in similar directions to improve packing density, particularly for smaller ROI such as Broca’s area seen previously. With five devices in the ACC-dlPFC, manual trajectory reached a mean of 182 bit/s while optimized reached 256 bits/s, a 40.7% improvement. Classification by SEPIO provides further insight into downstream potential for the trajectories. In manual trajectories, SEEG achieved a maximum accuracy of 14.9% and DiSc achieved 19.6%. Accuracy is understandably low given that the dlPFC contains 10,290 sources, totaling over four times ACC alone. In optimized trajectories, SEEG achieved a maximum accuracy of 25.6% and DiSc achieved 34.3%. Through optimization, classification improved by 4.7 points for SEEG and 15 points for DiSc.
